# Dentistry
*This article forms the introductory chapter of the new edition of Harris Principles and Practice of Dentistry about being issued by Lindsey and Blakiston, and is published by permission.


**Published:** 1871-07

**Authors:** P. H. Austen


AMERICAN JOURNAL

OF

DENTAL SCIENCE.

Vol. V. THIRD SERIES--JULY, 1871. No. 3.

### ARTICLE I.

### *Dentistry.**

By Professor P. H. Austen.

Dentistry is the Science and Art of Medicine, applied to the Dental Organs. Placed at the beginning of the alimentary canal, these organs hold an important relation to the digestive function, and through it to the entire body. They have also inseparable connections with the nervous, circulatory and respiratory systems. Hence, whilst their preservation constitutes an important art in medicine, the Science which teaches their structure, functions, diseases and treatment must necessarily be comprehensive. It must include those sciences which lie at the foundation of all medical art, and embrace so much of physical, mechanical and æsthetic science as its specific duties demand.

The Anatomy, Physiology and Pathology of dentistry differ in no respect from that taught in medical schools. The limits of a special text-book or curriculum of study, or a curtailment of the term of preparation may require the

* This article forms the introductory chapter of the new edition of Harris Principles and Practice of Dentistry about being issued by Lindsey and Blakiston, and is published by permission.

omission of some details, to give opportunity for a full exposition of others; but a dentist's knowledge of these fundamental sciences admits no limitation, except that imposed by mental capacity. A single volume upon the "Principles and Practice of Dentistry" must of necessity be rigidly eclectic in those sciences, each of which occupies many volumes for its full exposition; whilst it must give, in complete detail, all applications of science to its specific duties. Again, the eclecticism of teaching, both in the office and the college, is dependent upon the time over which it extends. Thus neither printed, oral, nor demonstrative systems of instruction can be taken as any correct measure of the amount of knowledge essential to professional excellence; for, in most cases, the knowledge thus gained is insufficient to give full value to the subsequent lessons of experience. The problem of professional education is one of difficult solution. While European systems seek to make "experts" of students, American systems are content to make them "experimenters." The Old-world regards three or four years of extra study a small matter, compared with the lives and welfare of the community: the New-world considers any risk preferable to such delay in entering upon the practical duties of life.

The Therapeutics of dentistry, unlike its anatomy, physiology and pathology, differs from that taught in the medical schools. It is Medical, Surgical and Prosthetic. In so far as it is a direction of medical science to the prevention, modification or removal, by medicinal and hygienic remedies, of the causes and effects of disease in the dental organs, it forms a part of a physician's practice, just as does the treatment of cerebral, cardiac, or pulmonary disease. In so far as it is an application of surgical skill to the extraction of teeth, the removal of tumors, to the treatment of fractures or to staphyloraphy, it is simply Oral surgery, involving only such knowledge and skill in the use of instruments as every surgeon must possess. But dental therapeutics includes a class of operations not taught in medical schools and not practised in the offices of physicians or surgeons: which, for their successful performance, require surroundings and appliances such as no other class of operations call for; demanding also an amount of time and special experience, which it is impossible for the general surgeon to devote to any one part of the body. Hence, by universal consent, this branch of therapeutics, under the name of Dental Surgery, is assigned to a special class of practitioners, who, like the oculist and obstetrician, perfect their art by limiting the sphere of its duties.

The prevailing and distinguishing feature of dental therapeutics is Prosthetics—the art of replacement: replacement of dental *structure*, in such manner and with such material as shall prevent further action of the destructive agencies; replacement of dental *organs*, by substitutes which shall physiologically restore impairment of function and æsthetically restore the natural expression of the face. The medical therapeutics and oral surgery of dentistry are insufficient to establish it as a distinct branch of medical art; whilst the operations of filing and regulating the teeth form a small proportion of its specific duties. It owes its extent to the universal liability of the teeth to decay and loss; it owes its difficulty, as an art, to the complex nature of the methods by which this loss and decay must be remedied. In other words Prosthetic Mechanism constitutes by far the largest and most difficult part of dentistry, makes it a distinct branch of the art of medicine, and gives to it the power to add as it does to health, comfort, and the enjoyment of life.

The physician, surgeon and dentist have necessarily many practical duties in common; but each has a clearly defined limitation of sphere, requiring specific direction of that general culture which all must possess. The Physician is a specialist; for, although he treats diseases which affect more or less the entire body, his therapeutics is restricted to hygiene and the materia medica, and there are many accidents, defects and pathological conditions, which are beyond the reach of his skill. Moreover, the Physician's specialty tends constantly to subdivision ; nor do we look for the most valuable contributions to medical science, except from those who apply themselves exclusively to some one class of diseases. Few minds can even approach that universality of genius, which characterized Hypocrates and John Hunter : hence devotion to a specialty of medical art detracts nothing from the position, which a man's education and talent entitle him to assume.

The Surgeon is a specialist, although few confine themselves to a practice purely surgical, except in cities and hospitals. Richerand correctly defined the specialism of surgical therapeutics as the "*quod in therapeia mechanicum:*" its well-known etymology conveys the same idea. Yet the element of mechanism and the necessity for the exercise of "hand-craft" enter, more or less, into all physical sciences. Astronomy, chemistry, pharmacy, microscopic analysis and modern medical diagnosis demand extreme accuracy of manipulation ; and all great discoverers in these sciences display the ability, not only to use, but also to invent and construct apparatus. The universal recognition of the great value of this element in every department of Physics has given the scientific world a more correct idea of the true dignity of high-educated mechanical skill—skill, without which the physician's art is crippled, surgery becomes impotent and dentistry has no existence.

The two departments of dental prosthetics, Structural and Organic, are usually classified as Operative and Mechanical dentistry. We have given preference, in this work, to the terms dental Surgery and dental Mechanics. Another classification is dentistry of the Chair and dentistry of the Laboratory. Each of these three classifications indicate prevailing characteristics, but all fail to point out the true basis both of the unity and the diversity of the two branches of dental practice. The editor does not feel at liberty to deviate, in the present volume, so widely from the author's arrangement: yet he may here suggest the following classification of dental therapeutics, or the Art of Dentistry. I. Medical. II. Surgical: (1. Oral surgery; 2. Dental surgery.) III. Prosthetic: (1. Structural; 2. Organic.)

As medicine and surgery are combined in the practice of the majority of medical men, so the two classes of prosthetic mechanism are usually practised together: but such practice, although, in a large number of cases, unavoidable, does not tend to the developement of highest excellence in either department. Certain details of the laboratory unfit the hand for some of the more delicate operations of structural prosthetics; whilst the engrossing and more remunerative duties of the chair almost inevitably lead to a hasty and negligent performance of laboratory work. The usual method of meeting this difficulty, that is by dividing the duties of organic prosthesis, cannot be too severely condemned. It is like requiring an artist to paint a correct portrait from verbal descirption: it ignores every principle of dental æsthetics, and its results are artificial dentures, so devoid of expression and individuality, as to mar the features they intended to adorn. But the prosthetic character of dentistry subjects it to a danger more serious than this unwise division of inseparable duties.

Scientific mechanism implies not only skill in construction, but judgement and purpose in application. Unfortunately a few months' use of tools enables one, who has natural aptitude in handling them, to producespecimens of workmanship, which are accepted as evidence of peculiar fitness for dentistry. If no early education has given habits of study, the fascinations of hand-work are permitted to engross time, that should be given to the harder and more distasteful head-work. The training, thus commencing and ending in mechanism, is discreditable not because of its mechanism, but because, being one-sided and partial, it necessarily fails to accomplish that which it promises. Such training may make dental laborers, tradesman, or artisans; but never dental artists, or scientific mechanicians: nor can the dentistry which they paactice be, in any respect, identified with that which we have defined as a branch of the art of medicine.

A preparation begun in pure science may end in correct practice, and the early habits of student-life may follow the professional man throughout his career; but a preparation, begun in practice will end there. The routine of professional duties often tempt the scholar to sink into the mere practitioner; it is rare indeed that one reverses the order of nature and sets aside the claims and emoluments of practice, to acquire slowly those habits of study so easily learned in youth. It requires the broadest literary and classical education of boyhood to counteract the necessarily narrowing influence of the professional studies of manhood; and it demands the largest possible infusion of purely scientific teaching, during professional pupilage, to correct the matter-of-fact influence of practice. In this lies the great error of American practical systems of education. They teach boyhood to take a utilitarian view of every lesson learned, and encourage young men to neglect studies in which they cannot see some prospective pecuniary value. It is the application to science and art, of that philosophy of life, which subordinates mind and body to the one idea of making a living; that spirit of trade, which regards classical study a waste of the years, in which plastic youth can best be moulded into the mercantile idea of Profit and Loss. Limitation, first in the amount of mental culture, secondly in its direction, is thus made to combine with the inevitable influence of all exclusive pursuit, whether as science or business; the result is a rapid increase, in all professions, of men whose vision is limited by the narrow horizon of their special occupation, and who possess none of the large-minded liberality, which is the outgrowth of a generous education. It is by such early restriction of thought and action within the narrow grooves of life's future pursuits that a merchant so often loses all power to enjoy the fruit of his toil, a physician is unknown beyond the sick-room, a surgeon contributes nothing to the cause of science, and a dentist holds no social position. This inevitable tendency of purely practical education was recognized by Lord Brougham when he recommended *Dante*, as a text-book, to an inquiring student of law.

The antagonism of trade and pure science is seen not only in the result of attempting to make all education utilitarian. It appears whenever, in professional life, the laws of barter come to be applied to brain-work and its products. Mercantile relations of cost and price are capable of definite adjustment, when applied to commodities of known values, enhanced by labor at given rates ; there are data also, upon which the speculative fluctuations of prospective supply and demand are based : so that in all bargains, buyer and seller may stand upon the ground of equal ability to judge these questions. But professional service is amenable to no such standard : the client cannot estimate the cost of his lawyer's pleading, nor can the patient know, until long afterwards, the full value of his physician's prescription. The conditions of honest barter are absent, for client and patient are alike dependent upon the integrity of the professional man ; hence professional bargaining is dishonorable, and inevitably leads to the rendering of a disreputable grade of service. The common practice of valuation by the visit or the hour is so manifestly unequal in its working, that it is only another proof of the impracticability of measuring science and art work by commercial standards.

The medical fee is a valuation of thought and skill, exercised for the preservation of life and health. On the part of the patient, it is considered a gratuity, by those who fail to perceive the elements of cost in such work ; a compensation by those who recognize an equivalent received ; an acknowledgement, by the few who refuse to believe that money can adequately reward such service. Viewed from the professional side, the fee has nothing to do with the quality of the service, nor does it enter into the mind of any right-thinking man, whilst rendering it. Mr. Ruskin says with great truth "it is impossible for a well-educated or brave man to make money the chief object of his thoughts; yet a healthy-minded man enjoys the honest winning of money, and will insist upon a fair valuation of his work. But with all brave men, the work is first and the fee second; whilst there is a vast class, ill educated, cowardly, and more or less stupid, with whom the fee is first and the work second."

All professions have suffered much by this perverted application of mercantile law to professional fees; but none so severely as dentistry. This is due to the prevalent idea, that the gold filling and the artificial denture are as legitimate objects of barter and contract, as any other tangible article of manufacture: whereas, in reality, they are no more so than the surgical operation, or the medical advice. When the dentist forsakes the vantage ground of a professional fee for, "services rendered," and condescends to bargain for the definite products of his skill, he at once destroys the professional character of his position. Not only does he lose caste; but in the class to which he has descended, the question of price invariably leads to considerations of cost, and the quality of his work, slowly perhaps, but surely deteriorates. The disastrous influence of vulcanite abundantly proves that, when cost of material is permitted to enter as an element in determining the value of scientific art-work, it inevitably degrades it; and the entire history of prosthetic dentistry shows that competition in price (the development of Mr. Ruskin's "fee first and work second") is fatal to all progress in art or advancement in science. The results of such competition are, to honest men, a life of slavish toil, with no time for self-improvement; to others, a deliberate slighting of work which destroys all the nobility of a man's nature. Dentistry, thus learned and thus practised, has no just claim to be called a profession; it has neither the liberality, generosity, nor culture, which men are accustomed to associate with professional life; and its pretentious claims serve only to call to mind the satire of Juvenal, "Scire volunt omnes, mercedem solvere nemo."

Dentistry, as a true science and art, is built upon the foundation of a generous early education, is enlightend by a complete medical course of instruction, is specially trained by a full term of practical pupilage, and recognizes no sliding-scale in the quality of the service it renders. Such dentistry will exercise influence in its own, and command respect among kindred, professions; for it becomes thus a curative work, second in importanceand extent of its usefulness to no specialty of the great Art of Healing.

